# The Impact of Comorbidities and Demographic Factors on Ejection Fraction

**DOI:** 10.3390/medicines11010001

**Published:** 2023-12-19

**Authors:** Cezara Andreea Soysaler, Cătălina Liliana Andrei, Octavian Ceban, Crina Julieta Sinescu

**Affiliations:** 1 Department of Cardiology, University of Medicine and Pharmacy “Carol Davila”, Emergency Hospital “Bagdasar-Arseni”, 050474 Bucharest, Romania; catalina.andrei@umfcd.ro (C.L.A.); crina.sinescu@umfcd.ro (C.J.S.); 2 Economic Cybernetics and Informatics Department, The Bucharest University of Economic Studies, 010374 Bucharest, Romania; octavianceban1995@gmail.com

**Keywords:** heart failure, gender differences, etiology, demographic factors, comorbidities

## Abstract

Heart failure (HF) presents an increasingly significant problem as the population ages. The cause of HF plays a significant role in determining treatment options and outcomes. It is worth noting that several studies have identified gender disparities in both morbidity and mortality, which may suggest differing causes of HF. The purpose of this research is to investigate the influence of various factors, including demographics and comorbidities, on ejection fraction (EF). The objectives of this study involve implementing preventive measures, ensuring timely diagnosis, and implementing interventions that target risk factors and specific comorbidities. These efforts aim to improve the prognosis for individuals affected by heart failure. The main method consists of linear regression. The demographic factors under scrutiny are gender and education, while the comorbidities of interest encompass valvulopathy, ischemia, smoking, obesity, high cholesterol, and diabetes. The main results consist of the fact that high education is associated with a 12.8% better EF on average, while among the factors with a negative role analyzed, ischemia is the most harmful, being 12.8% lower on average. Factors with a smaller impact are smoking, obesity, and high cholesterol. Diabetes does not seem to affect EF.

## 1. Introduction

Cardiovascular disease remains the leading cause of mortality worldwide, accounting for approximately 31% of global deaths, as reported by the World Health Organization [1].Globally, heart failure poses a significant public health challenge, with a prevalence of over 64.34 million cases (8.52 per 1000 inhabitants) in 2020 according to the Global Health Data Exchange registry, a database of health-related data maintained by the Institute for Health Metrics and Evaluation [2,3].

The etiology of heart failure is multifactorial and exhibits considerable variability based on factors such as sex, ethnicity, age, comorbidities, and environmental factors [4]. According to the Romanian Society of Cardiology—a member of the European Society of Cardiology—in 2018, it is estimated that 4.7% of Romanians aged over 35 years suffered from heart failure—about 650,000 patients. Every year, approx. 25,000 new patients are diagnosed with heart failure The prevalence of heart failure increases with age, reaching over 15% in population groups aged over 70 years [5,6]. Once diagnosed with heart failure, patients face a five-year survival rate of approximately 50% and a ten-year survival rate of around 10% [7].

A significant proportion of heart failure patients, specifically thirty-five percent, experience a high degree of disease severity and a markedly diminished quality of life. This decline is influenced by psychological factors, adverse treatment effects, and social limitations [8].

Noncardiac comorbidities play a crucial role in the progression of heart failure, and effectively managing them is essential for providing holistic care to heart failure patients. It is important to note that while conditions such as hypertension, diabetes mellitus, obesity, hyperlipidemia, and severe valvulopathy are known to be associated with the development of heart failure in the general population, their impact on the clinical outcomes and management of patients with established heart failure remains uncertain and poses significant challenges [9].

Recognizing and understanding the impact of social, behavioral, and comorbidity factors on the general population and particularly on individuals experiencing heart failure symptoms are crucial components for improving prognosis.

The purpose of this research is to investigate how specific factors, including demographics and comorbidities, impact left ventricular ejection fraction (EF) in patients with heart failure. The objectives of this study are focused on prevention, prompt diagnosis, and intervention targeting modifiable risk factors and specific comorbidities, all aimed at improving the prognosis of heart failure patients. Additionally, this study aims to identify the primary drivers of heart failure, which are later utilized to estimate disease progression and survival rates.

## 2. Materials and Methods

### 2.1. Methodology

By obtaining data through this research, the goal is to enhance the management of patients with heart failure, including treatment strategies and monitoring in primary care settings, with the ultimate aim of reducing morbidity and mortality. To achieve these objectives, an observational, prospective, comparative, and prognostic study was conducted, enrolling 197 patients aged 18 years and older. This study was carried out over a one-year period from October 2019 to October 2020 within the Hospital Cardiology Clinic of the emergency facility “Bagdasar-Arseni”. It should be mentioned that the patients included in this study were being treated with beta blockers, antiplatelet agents, nitrite statin, and oral antidiabetic drugs.

Informed consent was obtained from all subjects involved in this study. All participants were informed about their right to privacy and about the private storage and use of their data. This study did not present any potential psychological, social, physical, or legal harm to patients.

This study was conducted according to the guidelines of the Declaration of Helsinki and approved by the Ethics Committee of “Carol Davila” University of Medicine and Pharmacy, Bucharest, Romania (protocol code PO-35-F-03, date 12 July 2021).

Once a clinical diagnosis of heart failure (HF)is established, the underlying etiology plays a significant role in determining the appropriate treatment options and predicting the outcome. The presence of specific risk factors associated with HF development can impact both mortality and morbidity rates, and these factors may contribute to variations in clinical outcomes observed between genders [10,11].

We wanted to investigate how certain factors, demographics and comorbidities, affect EF. In this case, the factors are binary variables. The demographic factors are gender and education, and the comorbidities are valvulopathy, ischemia, smoker, obesity, high cholesterol, diabetes. We can get an idea of how these factors are correlated with EF from three perspectives. The first is to visualize the EF distribution compared at the factor level.

For example, how is the EF distributed for those with high education (the patients who graduated with at least bachelor’s degree) compared to those with low education (those who graduate only from high school). The second is to apply statistical tests to see if the means of these two distributions differ significantly. The third involves a more detailed analysis, using linear regression, where the dependent variable is always EF, and the independent variables are the previously mentioned factors, coded as dummy variables, since we are dealing with binary variables like is smoker, has obesity, has high cholesterol. A linear regression model was estimated for each factor. All data processing and fitting models were carried out using python programming language and classic packages for working with data, statistical tests, and model estimation such as pandas, numpy, scipy, statsmodels.

Following these regressions, one for each factor, different indicators were collected such as beta coefficients, *p*-value, std error. From the resulting table, we can better understand how these factors are correlated with EF.

### 2.2. Data

The sample consisted of 199 hospitalized patients with cardiovascular problems from the Hospital Cardiology Clinic of the emergency “BagdasarArseni” from Bucharest. Patients were divided according to gender, age, NYHA heart failure class and certain comorbidities. (Table 1 and Table 2). We did not have missing values.

## 3. Results

In the first phase of the research, we visualize the EF distributions compared at the factor level and associate a *p*-value, to check whether the means of the distributions differ significantly.

**Figure 1:**
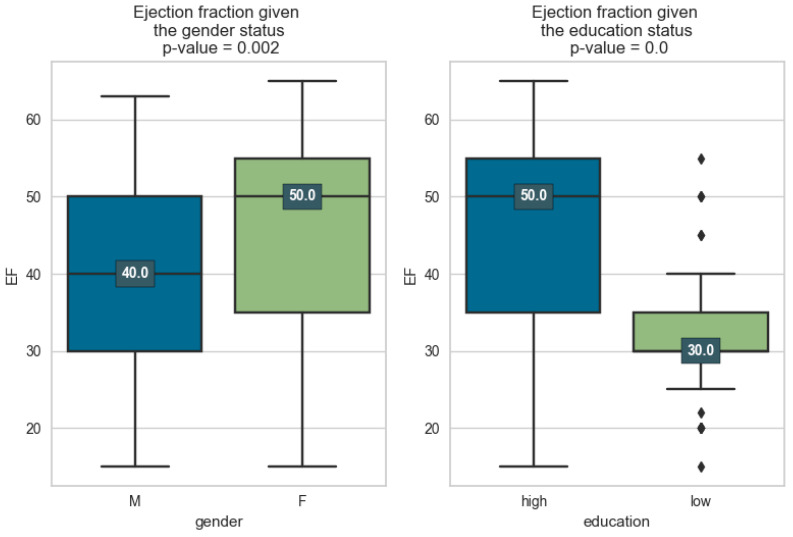
Distribution of ejection fraction grouped by demographic factors.

In Figure 1, it can be seen that we have significant differences both in the case of the gender factor and education. LV tends to be smaller for gender M. Previous epidemiological studies have provided evidence suggesting that premenopausal females have a lower incidence of cardiovascular disease (CVD) compared to age-matched males. On the other hand, the incidence and severity of CVD tend to increase after menopause. This lower incidence of CVD in women during their reproductive years can be attributed, at least in part, to the effects of estrogen.

It is interesting that in the case of education, the difference is much more obvious: those with low education have a much lower EF, which can be attributed to the fact that those with high education tend to give greater importance to prevention. Improved compliance with these self-care measures leads to better symptom control, reduced hospitalizations, and improved overall outcomes. Patient education also helps individuals develop a sense of self-efficacy, increasing their confidence in managing their condition and making informed decisions regarding their healthcare [12]. When patients are well informed and actively engaged in self-care, the need for hospital readmissions and emergency room visits can be minimized, resulting in cost savings for both the patients and the healthcare system [13].

In summary, patient education plays a vital role in heart failure management, promoting self-care, compliance, self-efficacy, and overall quality of life while reducing healthcare costs. It empowers patients to take an active role in managing their condition and leads to improved outcomes and well-being.

As we can see, comorbidities such as hypertension, dyslipidemia, obesity, and smoking can have a significant impact on the ejection fraction of the left ventricle. Arterial hypertension can cause an overload of the left ventricle, which in time can lead to left ventricular hypertrophy and affect ventricular relaxation. Both of these changes can result in a reduced ejection fraction.

Valvulopathies refer to diseases or abnormalities of heart valves. The severity of valvulopathies can influence the left ventricular ejection fraction (LVEF).

In severe valvulopathies, such as severe aortic stenosis or severe mitral regurgitation, the obstruction or regurgitation of blood flow through the valve is more pronounced, resulting in greater impairments in ventricular function and a lower LVEF. In contrast, mild valvulopathies may have less impact on the ejection fraction as the valve dysfunction is less severe. It is important to note that the relationship between valvulopathies and LVEF is complex and can vary depending on several factors, including the specific valve affected, the degree of valvular dysfunction, the duration of the condition, and individual patient characteristics.

The relationship between diabetes and its impact on systolic left ventricular function, specifically the left ventricular ejection fraction (LVEF), is a topic of ongoing research and discussion. The available evidence suggests that the association between diabetes and reduced LVEF may be influenced by the presence or absence of underlying coronary artery disease (CAD).

Diabetes is a well-established risk factor for the development and progression of CAD. In individuals with both diabetes and CAD, impaired blood flow can result in myocardial damage and affect left ventricular function, leading to a reduced LVEF.

**Figure 2:**
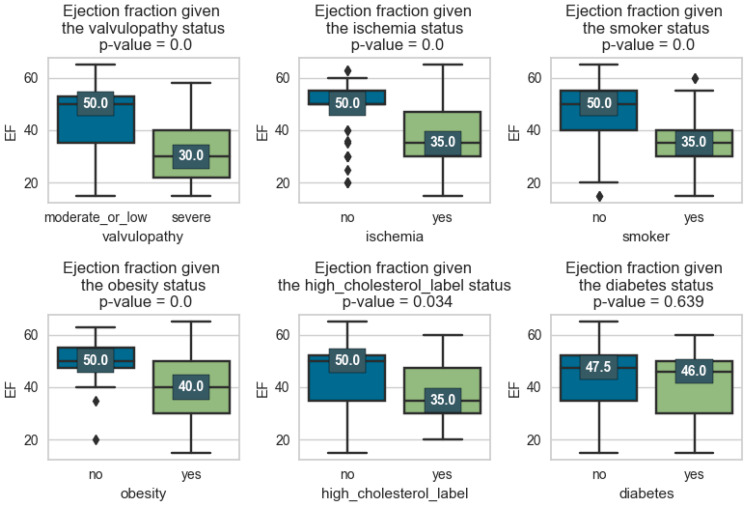
Comorbidity factors.

In order to determine in more detail how these factors influence EF and to measure their impact, eight linear models in parameters were estimated and the resulting indicators were collected as well. In the table below, the data from Figure 1 and Figure 2 above are added, i.e., the EF average for each factor, how many observations constituted that average, and what *p*-value makes the averages differ.

In the first column of Table 3, we have the binary variable X, and in the following, we have the average of EF for the two cases of the binary variable X (true or false). For example, for gender_male = 1 (true), we have an average EF of 39.1, which is calculated based on 91 observations. For gender_male = 0 (false), i.e., female patients, we have an average EF of 44.7, calculated on the basis of 106 observations. To see if these two averages differ significantly, we calculate the *p*-value which, in this example, is 0.002, so it differs significantly. It is noted that in all cases, there are significant differences in the means, with the exception of diabetes.

The beta1 coefficient shows how much, on average, EF increases when the independent variable is manifested. For example, a man has an EF of 5.585 units lower than that of a woman, and a patient with high education has an average EF of 12.8 units higher. It can be observed that in the case of all the factors, the *p*-value associated with beta 1 suggests that they are values significantly different from zero, with the exception of diabetes_yes, where there are no significant differences.

**Figure 3:**
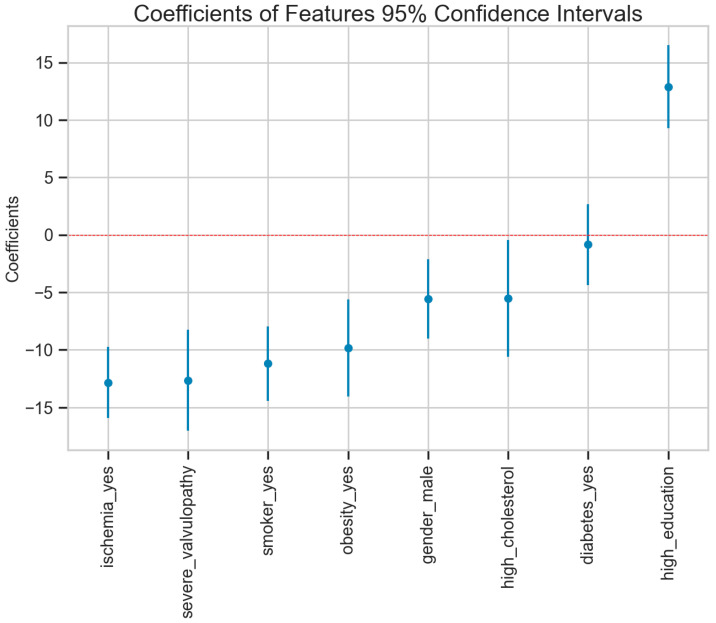
Estimations of the parameters and the confidence interval.

In the image above, (Figure 3) the importance of these factors and their error can be more clearly observed. In the case of education, a protective role is clearly seen; in the case of high cholesterol, it seems to have a negative influence, and in the case of the other factors, it is clearly seen that they are correlated with a large decrease in EF.

It is important to note that these comorbidities often coexist in patients and can have cumulative effects on the heart’s function and the ejection fraction. Additionally, individuals without any of these comorbidities generally have a lower risk of developing heart disease and, therefore, may have a better preserved left ventricular function.

Regular monitoring, early detection, and appropriate management of these comorbidities are crucial in preventing or minimizing the adverse effects on the ejection fraction and overall cardiac function. Lifestyle modifications, medication, and other interventions aimed at managing these conditions can help improve left ventricular function and reduce the risk of complications.

## 4. Discussion

The primary objective of this study was to conduct a comprehensive analysis comparing the disparities in heart failure outcomes between males and females, taking into consideration pertinent demographic variables such as gender and education, as well as relevant comorbidities including valvulopathy, ischemia, smoking, obesity, high cholesterol, and diabetes. The research demonstrated substantial disparities in ejection fraction values, both across genders and various levels of education, with male patients exhibiting notably lower ejection fraction levels.

Significantly discernible differences were observed concerning educational attainment, where individuals with lower educational backgrounds exhibited markedly reduced ejection fraction levels. This discrepancy is posited to stem from the tendency of those with higher education to prioritize preventive measures. Notably, patient education emerged as a pivotal factor in heart failure management, serving as a catalyst for empowering patients with essential self-care knowledge and skills. Moreover, education contributes to enhancing the patient’s quality of life by promoting self-management skills and fostering a sense of control over their condition. Patients who are knowledgeable about heart failure and its management are better equipped to actively participate in their own care, leading to improved physical and emotional well-being. Additionally, patient education tends to help prevention, and prevention would also bring economic benefits to the medical system. Unfortunately, we do not have data on the patients’ income. It would have been interesting to study income in this context of health and education. Education also probably brings a good income, which could lead to a healthier lifestyle.

This study underscores the critical role of patient education in enhancing compliance, self-efficacy, and overall quality of life among heart failure patients, concurrently mitigating healthcare expenditures. Comprehensive patient education encompasses vital aspects such as medication adherence, dietary modifications, fluid restriction, symptom recognition, adherence to exercise guidelines, and the significance of regular follow-up appointments. By imparting these crucial insights, healthcare providers can equip patients with the necessary tools to effectively manage their condition, thereby fostering improved health outcomes and reducing the burden on healthcare resources.

Comorbidities such as hypertension, dyslipidemia, obesity, and smoking exert significant influence on the ejection fraction of the left ventricle, a crucial parameter in assessing cardiac function. Arterial hypertension imposes an overload on the left ventricle, potentially culminating in left ventricular hypertrophy and compromised ventricular relaxation. Both of these alterations can lead to a reduced ejection fraction [14,15].

Obesity, recognized as a precursor to numerous cardiovascular risk factors including hypertension, dyslipidemia, and insulin resistance, induces left ventricular remodeling, impairs diastolic function, and diminishes ejection fraction [16,17]. Smoking, a substantial cardiovascular risk factor, elicits endothelial dysfunction, inflammation, oxidative stress, and vasoconstriction, all contributing to atherosclerosis development. Smoking-related coronary heart disease may result in myocardial infarction, subsequently impairing left ventricular function.

Elevated levels of LDL cholesterol and diminished levels of HDL cholesterol foster atherosclerosis, inducing ischemia and myocardial damage, thereby impacting the ejection fraction [18,19].

Valvulopathies encompass a spectrum of heart valve diseases and abnormalities. Several echocardiographic parameters change with age and are influenced by physical activity. The severity of valvulopathies significantly affects the left ventricular ejection fraction (LVEF), classified primarily into stenosis and regurgitation [20].

Valvular stenosis, occurring in aortic, mitral, pulmonary, or tricuspid valves, obstructs efficient blood ejection from the left ventricle, elevating ventricular pressure during contraction. Prolonged stenosis can lead to ventricular hypertrophy and potentially reduce LVEF. Valvular regurgitation, found in aortic, mitral, pulmonary, or tricuspid valves, results in an increased volume overload within the left ventricle due to backward blood flow during contraction. This condition may cause ventricular dilation and diminished LVEF [21].

The relationship between valvulopathies and LVEF is intricate, varying based on factors such as the specific valve affected, the degree of valvular dysfunction, the duration of the condition, and individual patient characteristics. Understanding these complexities is essential for a comprehensive evaluation of cardiac function and the development of effective management strategies.

Diabetes represents a widely recognized risk element in the advancement and escalation of coronary artery disease (CAD). In individuals with both diabetes and CAD, compromised blood circulation may give rise to myocardial injury, impacting the functioning of the left ventricle and consequently causing a decrease in left ventricular ejection fraction (LVEF).

However, in individuals with diabetes who do not have significant CAD, the impact on LVEF may be less clear. Some studies have suggested that diabetes itself may have a direct effect on left ventricular function, independent of CAD. Diabetes can contribute to structural changes in the heart muscle, such as left ventricular hypertrophy and fibrosis, which can affect systolic function and potentially lead to reduced LVEF.

It is important to note that research findings regarding the association between diabetes and LVEF have been inconsistent. Some studies have reported a significant reduction in LVEF among diabetics compared to non-diabetics, while others have not found a significant difference. The variation in study findings may be attributed to differences in study populations, methodology, duration of diabetes, control of other risk factors, and the presence or absence of CAD.

Overall, the impact of diabetes on systolic left ventricular function, specifically LVEF, may depend on various factors, including the presence of CAD. While diabetes is associated with an increased risk of developing cardiac complications, the exact relationship with LVEF is not yet fully elucidated, and further research is needed to better understand the underlying mechanisms.

Through a meticulous analysis of the aforementioned factors and their impact on heart failure, this study seeks to offer profound insights into the distinctive requisites and nuances essential for the prevention, diagnosis, and effective management of heart failure in both men and women.

By comprehensively grasping the gender-specific variations in heart failure, this research endeavors to inform the formulation of precise interventions and individualized treatment strategies. Such tailored approaches are crucial for enhancing outcomes among individuals of diverse genders, marking a significant advancement in the realm of cardiac healthcare.

The main limitations of this study are the small size and small diversity of the analyzed patients. Although it was sufficient to find significant results, it would have been interesting to see how these influences of comorbidities manifest themselves on different groups of people, taking into account age, sex, lifestyle, etc., which would have required much more data.

## 5. Conclusions

In conclusion, the escalating prevalence of heart failure (HF) within the aging demographic underscores its significance as a pressing public health concern. The underlying etiology of HF holds pivotal importance in shaping appropriate treatment modalities and subsequent outcomes. Research studies have consistently revealed variations in morbidity and mortality rates between genders, indicative of potential disparities in the root causes of HF. However, it is crucial to acknowledge that the existing body of knowledge concerning gender-specific differences in heart failure outcomes remains both limited and inconclusive.

It is imperative to emphasize the imperative for further research endeavors aimed at comprehensively unraveling the nuanced impact of gender on heart failure outcomes. Factors such as divergent underlying comorbidities, varying responses to treatment protocols, and unequal access to healthcare services may contribute significantly to the observed disparities. Continued and rigorous exploration of gender-specific distinctions in heart failure is indispensable, serving as the cornerstone for the development of tailored and effective treatment strategies. Such targeted approaches hold the promise of substantially enhancing outcomes for both men and women afflicted by this debilitating condition.

This study’s findings underscore the necessity of implementing precise and focused interventions that address the major risk factors associated with HF, while taking into careful consideration the wealth of gender-related data. This nuanced and informed approach is fundamental in effectively curbing the incidence of heart failure for individuals of all genders and represents a crucial stride toward improving overall cardiovascular health.

## Figures and Tables

**Table 1:** Counts for categorical data.

Count by Gender		Count by NYHA		Count by Smoker		Count by Hypertension		Count by Obesity	
F	106	IV	102	No	118	Severity_3	99	Yes	159
M	93	III	88	Yes	81	Severity_2	72	No	40
		II	8			Severity_1	14		
		I	1			No	14		

**Table 2:** Summary statistics for numerical variables.

	Age	Cholesterol	Triglyceride	EF
**count**	199	199	199	197
**mean**	70.38	174.13	126.95	42.14
**std**	10.73	53.8	124.47	12.59
**min**	40	24.1	25	15
**25%**	63	138.5	83	33
**50%**	71	163	100	46
**75%**	79	205	133.5	50
**max**	93	320	1222	65

**Table 3:** Linear regression results.

Variable X	Average of EF when Variable X Is True (E(Y|X = 1)	Count of EF when Variable X Is True(N(Y|X = 1)	Average of EF when Variable X Is False(E(Y|X = 0)	Count of EF when Variable X Is False(N(Y|X = 0)	*p*-Value Different EF Means	beta0	beta1	beta1 std Error	*p*-Value beta1	r Squared
gender_male	39.1	91	44.7	106	0.002	44.717	−5.585	1.76	0.002	0.049
high_education	45.4	147	32.5	50	0	32.52	12.888	1.85	0	0.199
severe_valvulopathy	31.6	33	44.3	164	0	44.256	−12.65	2.232	0	0.141
ischemia_yes	36.9	116	49.7	81	0	49.704	−12.85	1.58	0	0.253
smoker_yes	35.5	80	46.7	117	0	46.684	−11.196	1.647	0	0.192
obesity_yes	40.2	158	50	39	0	50.026	−9.836	2.145	0	0.097
high_cholesterol	37.4	27	42.9	170	0.034	42.894	−5.524	2.586	0.034	0.023
diabetes_yes	41.7	99	42.6	98	0.639	42.561	−0.844	1.798	0.639	0.001

## Data Availability

The data presented in this study are available on request from the corresponding author. The data are not publicly available, due to ethical restrictions.
